# From Polymorphisms to Phenotypes: *SMAD3* rs17293632 and *LTBP3* rs11545200 in Pediatric Inflammatory Bowel Disease

**DOI:** 10.3390/genes16121511

**Published:** 2025-12-16

**Authors:** Jan Brylak, Mariusz Szczepanik, Jan K. Nowak, Małgorzata Jamka, Aleksandra Glapa-Nowak, Aleksandra Banaszkiewicz, Andrzej Radzikowski, Anna Szaflarska-Popławska, Jarosław Kwiecień, Urszula Grzybowska-Chlebowczyk, Edyta Kawałkowska, Anna Wiernicka, Jarosław Walkowiak

**Affiliations:** 1 Department of Pediatric Gastroenterology and Metabolic Diseases, Poznan University of Medical Sciences, 60-572 Poznan, Poland; j.brylak@ump.edu.pl (J.B.); mszczepanik@ump.edu.pl (M.S.); jannowak@ump.edu.pl (J.K.N.); mjamka@ump.edu.pl (M.J.); glapa@ump.edu.pl (A.G.-N.); 2 Department of Pediatric Gastroenterology and Nutrition, Medical University of Warsaw, 02-091 Warsaw, Poland; abanaszkiewicz@wum.edu.pl (A.B.); andrzej.radzikowski@uckwum.pl (A.R.); 3 Department of Pediatric Endoscopy and Gastrointestinal Function Testing, Collegium Medicum in Bydgoszcz, Nicolaus Copernicus University in Torun, 85-094 Bydgoszcz, Poland; anna.szaflarska@cm.umk.edu.pl; 4 Department of Pediatrics, Faculty of Medical Sciences in Zabrze, Medical University of Silesia, 40-055 Katowice, Poland; jkwiecien@sum.edu.pl; 5 Department of Pediatrics, Faculty of Medical Sciences in Katowice, Medical University of Silesia, 40-055 Katowice, Poland; urszulachlebowczyk@wp.pl; 6 Department of Gastroenterology, Hepatology, Feeding Disorders and Pediatrics, The Children’s Memorial Health Institute, 04-730 Warsaw, Poland; e.kawalkowska@ipczd.pl (E.K.); a.wiernicka@ipczd.pl (A.W.)

**Keywords:** inflammatory bowel disease, Crohn’s disease, ulcerative colitis, pediatrics, *SMAD3*, *LTBP3*, TGF-β

## Abstract

**Background/Objectives**: Early-onset inflammatory bowel disease (IBD), including Crohn’s disease (CD) and ulcerative colitis (UC), frequently presents with a more severe clinical course. Genetic susceptibility, particularly involving the TGF-β signaling pathway, plays a key role in IBD pathogenesis. *SMAD3* and *LTBP3* encode crucial components of this pathway and have been implicated in IBD in previous genome-wide association studies. **Methods**: This study aimed to assess the clinical significance of the rs17293632 (*SMAD3*) and rs11545200 (*LTBP3*) polymorphisms in a pediatric IBD cohort. A total of 286 children (133 with UC and 153 with CD) were recruited from seven pediatric centers in Poland. Clinical data included age at diagnosis, inflammatory markers (CRP, albumin), growth indices (Z-scores for weight, height, and BMI), and treatment regimens. **Results**: The *LTBP3* rs11545200 minor allele was significantly associated with a younger age at diagnosis, poorer nutritional status during disease flares, and a more frequent use of infliximab—particularly in patients with UC. In CD, the *SMAD3* rs17293632 major homozygous genotype was associated with increased use of systemic corticosteroids, suggesting a more severe or treatment-resistant disease phenotype. **Conclusions**: The assessed polymorphisms in *LTBP3* and *SMAD3*, both involved in TGF-β signaling, are associated with clinical characteristics of pediatric IBD. These findings support the potential role of genetic variants as biomarkers for disease severity and treatment tailoring, contributing to the development of personalised therapeutic strategies in children with IBD.

## 1. Introduction

Inflammatory bowel disease (IBD) is a group of chronic autoimmune disorders primarily affecting the gastrointestinal tract [1], including Crohn’s disease (CD) and ulcerative colitis (UC). Both differ in their localisation and inflammatory pattern in the gastrointestinal tract. CD may involve any part of the gastrointestinal tract and presents with transmural, segmental (“skip”) inflammation, possibly causing fistulas or strictures. UC presents as inflammation of the rectum and colon, showing continuous inflammation of the superficial mucosa. CD carries a higher risk of malnutrition and perianal disease, while UC frequently presents with bloody diarrhea and carries a higher risk of colorectal cancer. While IBD mainly affects adults, up to 10% of cases are diagnosed during childhood [2,3], and this number is rising [4]. Notably, early-onset IBD cases often present a more aggressive course and require more intensive treatment [5,6]. This is partly related to the increased burden of predisposing genetic variants in pediatric IBD, which has led to numerous discoveries based on studies in children.

The global burden of IBD continues to rise, and these trends are thought to be linked to urbanisation, dietary changes, and other lifestyle-related factors [7]. In Poland, a recent estimate of the number of children with IBD was 3794 [8,9]. Although the incidence has shown stabilisation or even a slight decrease, the prevalence continues to rise in the entire population. The exact data for children in Poland are not yet available, but clinical practice suggests a growing prevalence.

While IBD is multifactorial, genetic predisposition interacts with environmental influences and the gut microbiome, shaping disease onset and progression [10,11]. Therefore, together with increasing prevalence, research into the genetics of IBD has been intensified. Genome-wide association studies (GWAS) identified over 300 loci associated with the risk of IBD [12,13], and it is thought to arise from an interplay between the exposome, predisposing genetics, and intestinal microbiota [4].

Even though extensive GWAS studies, and most recently multi-ethnic GWAS, have increased confidence in the genetics underlying IBD, which mostly underscore the role of the immune system, there is still an unmet need to understand the relationships between IBD genetics and the course of disease for personalised medicine [13,14,15]. Despite progress in understanding the genetic background of the disease risk, there is relatively little information on how genotype relates to the clinical course of IBD. Smaller, but more precisely phenotyped cohorts are useful to fill this gap in knowledge, as evidenced by one of our recent studies [16]. A genome-wide approach to answering analogous questions encounters sample size limitations, which are of lesser importance when the analysis is hypothesis-based and focuses on individual single-nucleotide polymorphisms (SNPs). Limited data are available in pediatrics, as most large cohorts have been based on the more prevalent IBD in adults. Therefore, studying the links between genotypes at pertinent genomic locations and clinical characteristics presents an interesting area of study.

In clinical practice, there is a need for biomarkers that can be assessed locally and cost-effectively, and real-time PCR makes this approach available. Likewise, the potential future scenarios of employing genetics in IBD differ. Polygenic risk scores [17,18] could be used to identify patients in need of lifestyle interventions to prevent IBD [19]. However, with additional phenotype data, future research may enable the identification of individuals with IBD at risk of a specific course. For instance, predicting the development of anti-drug antibodies (e.g., against infliximab) could open new avenues for early interventions. In such individuals, specific therapies or diagnostic studies could be adapted [19,20].

Notably, although the growth of knowledge about IBD genetics is rapid and holds many promises, clinicians still lack a comprehensive clinical analysis of the significance of many polymorphisms and their interactions in IBDs [21]. Addressing this problem requires studies with more focus on the phenotype, some of which may investigate individual key genes or molecular pathways. IBD arises from an overreactive tissue-damaging immune response that lacks sufficient counterregulatory mechanisms. These latter typically mitigate inflammatory pathways and support mucosal healing [22]. Dysregulated immune responses play a central role in IBD pathogenesis, particularly in pathways involved in inflammation resolution and tissue repair. Transforming growth factor-β (TGF-β) plays a central role in counteracting intestinal inflammation and supporting mucosal healing [23]. TGF-β is a multifunctional cytokine regulating immune tolerance, regeneration of mucosa, and remodeling of the extracellular matrix in the intestine. After ligand binding, TGF-β receptors activate the SMAD-dependent signaling cascade, in which receptor-regulated SMAD2 and SMAD3 proteins form a complex with SMAD4 and translocate to the nucleus to modulate the transcription of target genes, which are involved in tissue repair and immunosuppression [24]. This pathway suppresses the overactivation of effector T cells, promotes the differentiation of regulatory T cells, and improves the restoration of the epithelial barrier after injury [25].

**Figure 1:**
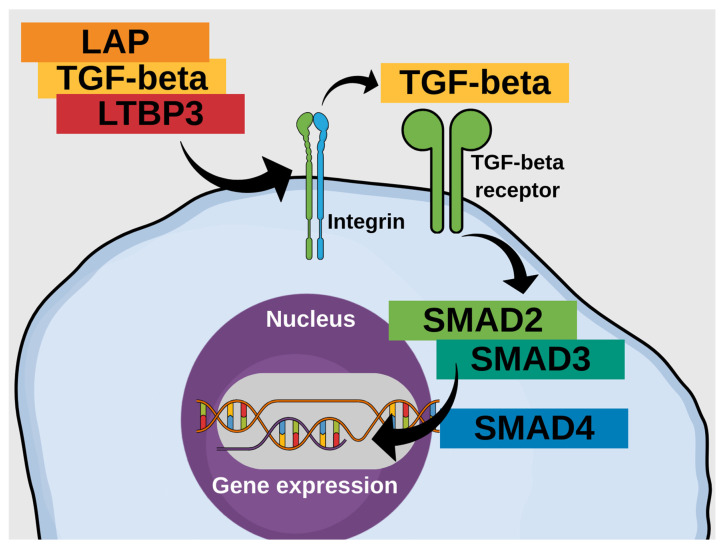
A simplified schematic of the canonical TGF-beta pathway illustrates the link between LTBP3 and SMAD3.

This knowledge contributed to the development of TGF-β2–enriched enteral nutrition formulas, which are now a cornerstone of exclusive enteral nutrition (EEN) in pediatric CD [26,27], showing comparable remission induction rates to corticosteroids in low-risk luminal disease [28,29,30,31]. The importance of the TGF-β pathway in the development of IBD was highlighted by the discovery of monogenic forms of IBD caused by mutations in this pathway [12,32]. *LTBP3* and *SMAD3* have garnered interest due to their reported associations with IBD in genome-wide association studies, particularly in pediatric populations [33,34,35]. The specific variants analysed here—*SMAD3* rs17293632 and *LTBP3* rs11545200—may affect pathways critical for mucosal immunity, tissue remodeling, and TGF-β signaling, all of which are central to IBD pathogenesis (Figure 1) [36].

The *SMAD3* gene encodes a signal transducer in the TGF-β signaling pathway, critical for regulating immune responses and maintaining gut epithelial integrity. The rs17293632 polymorphism, located in an intronic enhancer region, has been associated with altered *SMAD3* expression and increased susceptibility to both CD and UC [37,38]. It is thought to influence T cell differentiation and fibrotic responses, contributing to the chronic inflammation observed in IBD patients [39]. This SNP has also been implicated in a cardiovascular phenotype identical to Loeys-Dietz Syndrome (LDS type III) [40,41].

Similarly, *LTBP3* encodes a latent TGF-β binding protein which is essential for the activation and localisation of TGF-β within the extracellular matrix [42]. The rs11545200 polymorphism leads to a nonsynonymous amino acid substitution, potentially altering the protein’s interaction with TGF-β and impairing immune regulation. It is related to the homeostasis of connective tissue [43], while knowledge regarding its relationship to IBD is limited. However, as mentioned previously, its role is critical in the TGF-β pathway, which is relevant for IBD development.

Modern genotyping techniques include next-generation sequencing, which offers high quality but requires cost and time; genotyping microarrays that are also more costly and need to be used in batches; and real-time PCR genotyping, which is easily scaled and adopted in clinical conditions. This study applies real-time PCR because of its availability, precision, and ease of translation.

The significance of these SNPs in diagnosis lies in their predictive value for disease susceptibility and progression. Genotyping for *SMAD3* rs17293632 and *LTBP3* rs11545200 may enhance early identification of at-risk children and facilitate personalised treatment strategies, particularly when integrated into polygenic risk scoring. This study, therefore, aimed to assess the clinical significance of these variants in children with IBD, focusing on associations with disease phenotype and therapeutic needs.

## 2. Materials and Methods

### 2.1. Bioethics and Recruitment

Our study was conducted using blood samples collected between 2016 and 2019 from seven pediatric university hospitals in Poland, located in Poznań (*n* = 1), Warsaw (*n* = 2), Wrocław (*n* = 1), Zabrze (*n* = 1), Katowice (*n* = 1), and Bydgoszcz (*n* = 1). Inclusion criteria constituted (i) a diagnosis of IBD following current guidelines, (ii) patient age between 3 and 18 years, and (iii) no life-threatening symptoms. The diagnosis of IBD was established in all participating centers according to the revised ESPGHAN Porto criteria [44]. Endoscopy of the lower gastrointestinal tract was performed under general anesthesia. The final IBD diagnosis relied on a combination of clinical, endoscopic, and imaging findings. Informed consent was obtained from the parents or legal guardians of all participants, as well as from adolescents aged 16 years or older [45]. The study received formal approval from the Bioethical Committee at Poznan University of Medical Sciences (approval number 960/15, with amendment from December 2020) and adhered to the principles of the Declaration of Helsinki [46].

### 2.2. Clinical Characteristics

The data included the age at diagnosis and study inclusion, gender, the type of IBD (UC or CD, excluding IBD-unspecified), and various clinical parameters assessed at diagnosis and during the most severe flare-up (Table 1). These parameters included serum C-reactive protein (CRP), albumin level, the disease activity measured using the Pediatric Ulcerative Colitis Activity Index (PUCAI) for UC, or the Pediatric Crohn’s Disease Activity Index (PCDAI) for CD, and Z-scores for body mass, height, and body mass index (BMI) based on local reference standards. CRP or albumin concentrations were available for a considerable proportion of patients at diagnosis and/or worst flare, depending on disease duration and retrospective data availability. Disease severity was evaluated based on treatment modalities, including systemic steroids, azathioprine, methotrexate, ciclosporin, and biologics (infliximab and adalimumab) (Table 2). Additional data included the time from diagnosis to the initiation of biologic therapy, the age at which biologic treatment began, the necessity for IBD-related surgery, the number of hospitalisations for IBD flare-ups, and, for patients with disease duration over one year, the annual frequency and total days of hospitalisation due to flare-ups.

Older adolescent patients in the transition period to adult care were fully followed up by pediatric centers, and these centers had no opportunity to treat or clinically follow the patients once they reached the age of 18 years. However, in some cases, it was hypothetically possible to obtain missing data shortly after this period. When data were missing, we avoided imputation and preferred to only use complete data. This results in lower numbers of patients included in specific analyses and reduces power, but does not reduce the specificity of the results.

### 2.3. Genotyping

After obtaining samples, which were stored at −20 °C, DNA was extracted from whole blood collected in EDTA tubes using a microcolumn-based kit (Blood Mini, A&A Biotechnology, Gdynia, Poland). The concentration and purity of DNA were assessed using a NanoDrop Lite spectrophotometer (Thermo Fisher Scientific, Waltham, MA, USA). Genotyping employed a TaqMan probe alongside the TaqMan Genotyping Master Mix (Thermo Fisher Scientific Inc., Waltham, MA, USA). Real-time PCR reactions were performed using the CFX96 thermocycler system. The temperatures were 60 °C for 30 s. And 95 °C for 5 min., followed by 40 cycles of 95 °C for 15 s, 60 °C for 1 min., and plate read. Fluorescence was analysed using Bio-Rad software (version 1.6; Bio-Rad, Hercules, CA, USA). Allelic discrimination analysis was performed manually by analysing the data in R to visualise optimal clusters. Data with insufficient fluorescence on both channels were excluded from the study. The call rate was 87.2% for the *LTBP3* polymorphism and 96.1% for *SMAD3*. Hardy–Weinberg equilibrium testing was done using the Hardy–Weinberg R package (version 1.7.9); the chi-square test for *LTBP3* and Fisher’s exact test for *SMAD3* (because of low MAF = 5.8%) were performed. The resulting *p*-values were 0.7255 for the *LTBP3* polymorphism and *p* = 0.0061 for the *SMAD3* SNP. Because the call rate for *SMAD3* was acceptable and because clustering was clear, it may be speculated that stratification may have contributed to the lack of Hardy–Weinberg equilibrium in the data for *SMAD3*. The obtained data were further analysed in R (version 4.5.1), as well as PQStat (version 1.8.6.122; PQStat Software, Poznan, Poland).

### 2.4. Statistics

Descriptive statistics were calculated for all variables. Comparisons between groups were conducted using the Mann–Whitney U test for continuous variables and Fisher’s exact test for binary (0/1) variables. A *p*-value lower than 0.05 was considered statistically significant [47]. The distribution of *SMAD3* and *LTBP3* gene polymorphisms was analysed by comparing the major homozygous genotype versus all other genotypes, and the minor homozygous genotype versus all other genotypes.

## 3. Results

### 3.1. Clinical Characteristics of the Study Cohort

The study cohort included 286 children and adolescents diagnosed with either UC (*n* = 133) or CD (*n* = 153). The median age at diagnosis was comparable between groups: 12.0 years (IQR: 7.8–14.7) for UC and 12.6 years (IQR: 10.4–14.3) for CD. However, patients with UC were included in the study at a younger age (median 14.8 years vs. 15.3 years), despite having similar disease durations at the time of analysis (median 2.0 years vs. 2.3 years, respectively). Females accounted for 49.3% of the UC group and 42.2% of the CD group.

### 3.2. Laboratory and Nutritional Parameters

Inflammatory markers were significantly higher in CD. Median CRP levels at diagnosis and during the worst disease flare were notably elevated in CD (15.0 and 16.8 mg/L, respectively) compared to UC (2.4 and 2.6 mg/L). Also, albumin levels were lower in CD at both time points (3.8 and 3.9 g/dL vs. 4.1 and 4.2 g/dL in UC), also suggesting a more severe inflammation in CD.

Anthropometric measurements reflected this trend, with pediatric patients with CD exhibiting more pronounced nutritional deficits. At diagnosis, median Z-scores for body mass, height, and BMI were lower in CD (−0.8, −0.3, and −0.8, respectively) than in UC (−0.5, 0.0, and −0.5, respectively), and this disparity persisted during the most severe flare.

### 3.3. Disease Activity and Treatment Characteristics

Disease activity scores also highlighted differences. PUCAI increased from 45 at diagnosis to 55 during the worst flare in UC, while PCDAI rose from 30 to 40 in CD over the same course.

Both groups received a range of treatments, which could include systemic corticosteroids, azathioprine, methotrexate, ciclosporin, and biologics. During the 2016–2019 study period, when access to biologics in Poland was still limited, this type of therapy started sooner in CD (median 12.3 months post-diagnosis) compared to UC (14.3 months after disease onset). However, patients with UC began biologic treatment at a younger age overall (median, 11.0 years vs. 13.6 years in CD), likely reflecting different clinical criteria for initiating biologics between UC and CD. Infliximab was used more frequently than adalimumab in both groups.

Systemic corticosteroids and azathioprine were more commonly used in UC, which also showed a higher frequency of IBD-related surgical interventions. Hospitalisations for disease flares were more frequent in UC (median 2 per patient) compared to CD (median 1). However, annualised hospitalisation rates and their relative duration were comparable between groups (UC: 0.7 hospitalisations and 4.8 days/year; CD: 0.5 hospitalisations and 4.3 days/year).

### 3.4. Associations of LTBP3 rs11545200

For the *LTBP3* rs11545200 polymorphism in the overall IBD cohort, an association was observed with selected clinical parameters (Table 3 & Supplementary Table S1). The presence of the minor allele was linked to a younger age at IBD diagnosis (*p* = 0.0266) and an increased frequency of infliximab use (*p* = 0.0267). Associations were also noted between the *LTBP3* genotype and Z-scores for body mass and BMI, with the most reliable trend—based on the number of patients—indicating poorer nutritional status during the most severe disease flare in patients with the rs11545200 polymorphism.

In the UC subgroup, where the statistical power was lower, no significant association was found between the polymorphism and age at diagnosis. However, the previously described association with infliximab use remained substantial (*p* = 0.0378), and an analogous association with adalimumab use was also identified (*p* = 0.0377). Additionally, the need for more intensive treatment in this group was supported by an association between the polymorphism and the use of ciclosporin (*p* = 0.0058).

In contrast to UC, the *LTBP3* polymorphism in the CD subgroup was associated with disease duration; patients with the major homozygous genotype had a shorter disease duration at the moment of inclusion. This may have influenced other clinical parameters in this group. Although a trend toward earlier diagnosis in carriers of the minor allele was observed, it did not reach statistical significance (*p* = 0.0546). Poor nutritional status during the worst disease flare was also found in UC in the major homozygous group (*p* = 0.0075 for body mass; *p* = 0.0050 for BMI). This was accompanied by a higher frequency of hospitalisations due to disease flares (*p* = 0.0059). However, this finding may have been confounded by differences in the duration of the disease. No associations were found between the *LTBP3* polymorphism and the type of treatment used in CD.

### 3.5. Associations of SMAD3 rs17293632

Regarding the *SMAD3* rs17293632 polymorphism, the major homozygous genotype was marginally more frequent in CD compared to UC (*p* = 0.0476). One notable difference was observed: a higher frequency of systemic corticosteroid use among patients with the major homozygous genotype.

No statistically significant associations were identified between *SMAD3* rs17293632 and disease course in the UC subgroup. In CD, patients with the minor heterozygous genotype (*n* = 6) appeared to be younger at the time of inclusion (*p* = 0.0242). Additionally, the need for systemic corticosteroids (*p* = 0.0125) and methotrexate (*p* = 0.0333) may have been associated with the *SMAD3* genotype, although detailed post hoc analyses did not confirm these associations. It is important to note that the results have not been corrected for multiple testing and should therefore be interpreted with caution.

## 4. Discussion

This multicenter genetic association study evaluated the clinical significance of two polymorphisms, *SMAD3* rs17293632 and *LTBP3* rs11545200, in a cohort of 286 pediatric patients diagnosed with UC or CD. Genotypes were linked to several clinical parameters, including age at diagnosis, markers of inflammation, growth indicators, disease severity scores, treatment modalities, and hospitalisation rates. The findings may provide insights into how such polymorphisms influence the course of the disease and could help identify patients who might benefit from specific therapeutic strategies.

Because of recruitment at university hospitals, the presented cohort of children with IBD is more representative of the more frequently hospitalised population, which is characterised by more severe disease. This number of patients constituted approximately 8% of all children with IBD in Poland [9]. Also, this group was recruited across the whole country. Access to biological treatment started to improve during the course of study.

The most statistically and clinically significant findings include a strong association between the *LTBP3* rs11545200 minor allele and a younger age at diagnosis, as well as an increased need for future biologic therapy, particularly in UC patients.

This association suggests that genetic variation in extracellular matrix regulation, where *LTBP3* plays a critical role, may modulate disease severity or therapeutic response. There are sparse data regarding the role of *LTBP3*; however, Zhao et. al. showed that common *LTBP3* variants are functionally relevant and can influence the integrity of connective tissue [43]. Another study found an impact of *LTBP3* mutations on the extracellular matrix function in ophthalmic pathology [48]. Therefore, SNPs in this gene seem to have the potential for systemic influence.

*LTBP3* encodes a latent TGF-β binding protein involved in the storage, secretion, and activation of TGF-β, a cytokine essential for maintaining intestinal immune tolerance and mucosal healing [23]. Disruption of this balance may lead to exaggerated inflammation and poor response to first-line treatments (e.g., 5-ASA and steroids), necessitating early biologic therapy [22].

The observed trend toward poorer nutritional outcomes and more frequent hospitalisations in carriers of the risk genotype further supports the hypothesis that this variant of *LTBP3* is a proxy for a more severe disease phenotype.

Secondly, in CD patients, the *SMAD3* rs17293632 major homozygous (CC) genotype was more common. It showed an association with systemic corticosteroid use, suggesting a role in modulating disease activity or therapeutic resistance, hence an association with more severe or refractory disease.

*SMAD3* is a central intracellular mediator of the TGF-β signaling pathway [34,49]. Polymorphisms in the *SMAD3* gene can alter the transcriptional response to TGF-β, potentially contributing to exaggerated immune activation and impaired mucosal repair in IBD [50].

The association with more frequent corticosteroid use in major homozygous variants of *SMAD3* rs17293632 suggests that this variant may predict responsiveness to standard immunosuppressive therapies and, in the future, might become a marker for personalised treatment planning. The usefulness of assessing variants of *SMAD3* was also supported by a previous study provided by O’Donnell et al. [36].

Existing studies suggest a direct correlation between *SMAD3* genetics and IBD [33]. Animal models and pathway analyses further substantiate this connection; Fowler et al. identified that homozygous *SMAD3* rs17293632 increases the risk of surgery in CD [51], while another study provided information indicating that this SNP does not influence the course of the disease in CD after terminal ileal resection [52]. Moreover, GWAS have linked the *SMAD3* locus, specifically SNP rs17293632, to an increased risk of CD, underscoring the clinical relevance of this pathway in intestinal inflammation and repair [53].

According to the Open Targets Platform, rs17293632, despite no direct impact on protein structure, is a *SMAD3* transcript usage-, splicing- and expression-quantitative trait locus (tuQTL, sQTL & eQTL). It was also associated with asthma by GWAS. *LTBP3* rs11545200 is an LTBP3 protein-QTL, as well as an sQTL for *MALAT1*, and a single-cell expression QTL (sceQTL) for *LTBP3* in CD4+ alpha-beta T cells. As per Ensemble Variant Effect Predictor, it is a missense *LTBP3* variant (A438V) with moderate impact on the protein. Therefore, potential mechanisms of impact on IBD pathogenesis exist for both investigated variants.

Although not examined in this study, other variants in the TGF-β signalling pathway have been linked to IBD. Firstly, *TGFBR1* and *TGFBR2* polymorphisms appear to be associated with defective receptor transmission and impaired immune regulation [54]. Secondly, variants in *SMAD7*, which inhibit TGF-β signaling, have been correlated with non-response to infliximab [55]. Lastly, *CTGF* gene SNPs, such as those reported by Burke et al., influence intestinal fibrosis in CD [52]. While these specific genes were not included in our analysis, they remain relevant targets for future genetic screening and mechanistic research.

The TGF-beta signaling pathway exerts important effects in IBD through inflammation and mucosal healing. The cascade starts with latent TGF-beta, which is associated with LTBP—the subject of this study—and latency-associated peptide. The release of TGF-beta is possible due to the activity of integrins (αvβ6 and αvβ8), and other factors such as extracellular proteases and physical traction. Presence of antibodies against αvβ6 precedes the diagnosis of UC by several years [56]. The integrin αvβ8 was also implicated in IBD, because its deficiency promotes colitis in a murine model [57]. Furthermore, rare *ITGAV* mutations cause colitis [58]. Active TGF-beta may bind the TGF-beta receptor type II, which starts a sequence of events involving TGF-beta receptor type I and leads to activation of SMAD2 and SMAD3 (the subject of this study). These two proteins can bind SMAD4, after which the complex can translocate to the nucleus, thus affecting cellular function through interaction with transcription factors, which are tightly regulated. Importin 8 (IPO8) plays an important role in this translocation, and its defects may also affect immunity [59]. Importantly, genes that are targeted by the SMAD complex include FOXP3, which is crucial for the development of tolerogenic T regulatory cells, and IL10, which is the key anti-inflammatory cytokine. Defects in *FOXP3* and IL10 receptor genes are widely recognised as causes of early-onset IBD [12]. Of course, TGF-beta has broad impacts spanning from embryogenesis to tissue repair [60], with potential relevance to fibrosis in CD [61]. This latter function is associated with healing. TGF-beta may also have SMAD-independent effects by acting through non-canonical routes involving NFkB, MEK/ERK, as well as mTORC and STAT, resulting in a very broad reach and high potential of influence on immune functions. This being summarised, TGFB1 deficiency causes VEO-IBD, while Loeys–Dietz syndrome (caused by mutations in various genes of TGF-beta signaling) involves immune dysregulation with intestinal inflammation. Thus, TGF-beta is directly relevant to IBD, and the current study investigates two of the key genes of this pathway.

Multi-omics approaches have significantly advanced our understanding of how the TGF-β signaling pathway contributes to the pathogenesis of IBD. Genomic studies, particularly GWAS, have consistently identified key genes such as *SMAD3*, *SMAD7*, and the TGF-β receptors (*TGFBR1* and *TGFBR2*) as associated with increased susceptibility to IBD [34]. At the transcriptomic level, researchers have observed that expression levels of genes of the TGF-β pathway vary depending on the severity of the disease and the patient’s response to therapy, suggesting a role in both pathophysiology and treatment response [62,63]. Epigenetic modifications, particularly DNA methylation of genes such as *SMAD3*, further modulate gene expression and may serve as predictive biomarkers for treatment outcomes [64]. These molecular insights are supported by findings from animal models, in which mice with impaired TGF-β signalling spontaneously develop colitis, providing strong evidence for a direct, causative role of this pathway in the development of intestinal inflammation [65,66]. As our understanding of the TGF-β pathway advances, we gain a deeper insight into the pathomechanism of IBD, laying the groundwork for the development of biomarkers that may leverage genetic insights. The *SMAD3* rs17293632 polymorphism was found in the IBD polygenic risk score by Monti et al. [67] and in the combined IBD GWAS data from the International Inflammatory Bowel Disease Genetics Consortium.

If replicated in larger cohorts, genotypic information related to *LTBP3* or *SMAD3* could contribute to communication with families by clarifying potential disease trajectories or the likelihood of requiring advanced therapies. Such data, combined with clinical indicators, may help personalise the discussion on treatment options and long-term management strategies.

Some of the characteristics of this study result in effects that require discussion. The sample size is moderate for genetic analysis, precluding effective epistasis analysis. The dichotomisation of genotypes may lead to oversimplification of associations that may still show evidence of more discretised associations. Even the strongest of the obtained results would become insignificant after Bonferroni or Holm–Bonferroni correction for multiple testing, which must be taken into account when considering potential clinical applications of the results. The lack of a healthy control group presents a limitation, but does not significantly alter the interpretation of variants as disease-modifying, because it is known that some factors predisposing to IBD do not need to produce more severe disease.

## 5. Conclusions

This study demonstrates that TGF-β-related polymorphisms rs11545200 of *LTBP3* and rs17293632 of *SMAD3*, both of which relate to the TGF-β signaling pathway, are associated with the clinical course of pediatric IBD.

The presence of the *LTBP3* rs11545200 minor allele was significantly associated with earlier disease onset, greater nutritional impairment, and an increased need for biologic and immunosuppressive therapies, particularly in UC. In CD, the *SMAD3* rs17293632 major homozygous genotype was associated with a more frequent use of systemic corticosteroids, potentially reflecting more severe or refractory disease.

Overall, the study supports a modulatory role of TGF-β pathway-related genetic variants in pediatric IBD phenotypes. The findings suggest the potential for incorporating such variants into predictive models for disease severity and treatment responsiveness. Research into links between genetics and the course of IBD is likely to continue yielding new data, with potential for clinical applications.

## Figures and Tables

**Table 1:** Characteristics of assessed patients with ulcerative colitis (UC) and Crohn’s disease (CD).

	UC		CD	
Analysed Variables	*n*	Median (Q1–Q3)	*n*	Median (Q1–Q3)
Age at diagnosis [years]	133	12.0 (7.8–14.7)	153	12.6 (10.4–14.3)
Age at inclusion [years]	127	14.8 (11.6–16.9)	153	15.3 (13.6–17.1)
Duration of the disease [years]	122	2.0 (0.4–3.8)	151	2.3 (0.8–4.1)
Female		49.3%		42.2%
CRP at diagnosis [mg/L]	124	2.4 (0.6–11.6)	149	15.0 (2.4–31.2)
CRP at worst flare [mg/L]	108	2.6 (0.8–12)	130	16.8 (3–37.1)
Albumin at diagnosis [g/dL]	114	4.1 (3.7–4.4)	136	3.8 (3.5–4.2)
Albumin at worst flare [g/dL]	103	4.2 (3.8–4.4)	125	3.9 (3.5–4.2)
Mass Z-score at diagnosis	126	−0.5 (−1.1–0.3)	144	−0.8 (−1.4–0.1)
Height Z-score at diagnosis	125	0 (−0.7–0.8)	144	−0.3 (−1.4–0.5)
BMI Z-score at diagnosis	125	−0.5 (−0.9–0.2)	144	−0.8 (−1.4–0)
Mass Z-score at worst flare	108	−0.5 (−1–0.4)	125	−0.8 (−1.4–0.2)
Height Z-score at worst flare	109	0 (−0.7–0.6)	126	−0.4 (−1.3–0.3)
BMI Z-score at worst flare	108	−0.6 (−1–0.2)	125	−0.8 (−1.4–0.1)
PCDAI at diagnosis	2	35 (32.5–37.5)	136	30 (22.5–47.5)
PUCAI at diagnosis	115	45 (30–60)	3	40 (37.5–52.5)
PCDAI at worst flare	1		120	40 (30–52.5)
PUCAI at worst flare	103	55.0 (36.3–65)	2	56.3 (46.9–65.6)

BMI—body mass index, CRP—C-reactive protein, PCDAI—Pediatric Crohn’s Disease Activity Index, PUCAI—Pediatric Ulcerative Colitis Activity Index.

**Table 2:** Therapeutic management and disease outcomes in patients with ulcerative colitis (UC) and Crohn’s disease (CD).

	UC		CD	
Analysed Variables	*n*	Median (Q1–Q3)	*n*	Median (Q1–Q3)
Systemic steroids	134		154	
Azathioprine	133		154	
Methotrexate	133		154	
Ciclosporin	133		154	
Biologics	134		154	
Infliximab	131		153	
Adalimumab	131		153	
Months from diagnosis to biological therapy	34	14.3 (9.2–27)	70	12.3 (6.1–26)
Age at first biological therapy	35	11.0 (6.4–15.2)	70	13.6 (12.4–15)
IBD-related surgery	134		154	
Times hospitalised for IBD flare	114	2 (1–3.8)	130	1 (1–2)
Hospitalisations for IBD flare per year	71	0.7 (0.3–1.4)	95	0.5 (0.2–0.9)
Days hospitalised for IBD flare per year	71	4.8 (1.6–9.2)	95	4.3 (1.1–7.3)

**Table 3:** Summary of main findings from the analysis of polymorphisms in *LTBP3* rs11545200 and *SMAD3* rs17293632.

Gene/SNP	Subgroup	Associated Clinical Parameters	Allele Type, Homozygous vs. Remaining Genotypes	*p*-Value
*LTBP3* rs11545200	IBD	Age at diagnosis	minor	0.0266
	IBD	Body mass Z-score at worst flare	minor	0.0045
	IBD	BMI Z-score at worst flare	minor	0.0008
	IBD	Biologics use	minor	0.0323
	IBD	Infliximab use	minor	0.0267
	UC	Infliximab use	minor	0.0378
	UC	Adalimumab use	minor	0.0377
	UC	Ciclosporin use	minor	0.00577
	CD	Disease duration	major	0.0155
	CD	Body mass Z-score at worst flare	major	0.0075
	CD	BMI Z-score at worst flare	major	0.005
	CD	Hospitalisations for flares	major	0.0059
*SMAD3* rs17293632	IBD	Systemic corticosteroid use	major	0.0137
	CD	Age at inclusion	minor	0.0242
	CD	Corticosteroid use	heterozygous	0.0125
	CD	Methotrexate use	minor	0.0333

CD—Crohn’s disease, SNP—single-nucleotide polymorphism, UC—ulcerative colitis.

## Data Availability

The data presented in this study are available from the corresponding author upon reasonable request.

## Supplementary Materials

The following supporting information can be downloaded at: https://www.mdpi.com/article/10.3390/genes16121511/s1, Table S1: Detailed statistical outcomes for *LTBP3* rs11545200 and *SMAD3* rs17293632.
